# A longitudinal study of the mental health of autistic children and adolescents and their parents during COVID-19: Part 2, qualitative findings

**DOI:** 10.1177/13623613221086997

**Published:** 2023-01

**Authors:** Kathryn Asbury, Umar Toseeb

**Affiliations:** University of York, UK

**Keywords:** autism, COVID-19, longitudinal, mental health, mixed-methods, qualitative, quantitative, special educational needs

## Abstract

In Part 1 of this UK-based study, across four timepoints between March and October 2020, autistic children and young people showed higher levels of parent-reported depression and anxiety symptoms than those with other special educational needs and disabilities. In this study, we draw on qualitative data from 478 parents/carers of autistic pupils and those with other special educational needs and disabilities to conduct a longitudinal qualitative content analysis examining stability and change in the mental health of these young people, and their parents/carers, during the first 6 months of the COVID-19 pandemic. Worry and psychological distress were dominant categories at all timepoints and we noted that, in line with quantitative findings, worry in autistic pupils stayed stable over time but decreased for those with other special educational needs and disabilities. The third dominant category was wellbeing and we saw evidence that removing demands, especially the demand to attend school, was a driver of wellbeing for a significant minority of pupils, particularly autistic pupils, and their parents/carers. Overall, we observed no differences in mental health experiences between the two groups of parents, also mirroring quantitative findings.

**Lay abstract:**

We know that autistic children and young people, and their caregivers, are at increased risk of mental ill health. We asked whether the first 6 months of COVID-19 exacerbated that risk, and whether the implications were different for autistic pupils and their caregivers, than for those with other special educational needs and difficulties. In a linked paper, we found that caregivers of autistic pupils reported higher levels of depression and anxiety symptoms in their children than parents of children with other special educational needs and difficulties (Toseeb & Asbury, 2022). For pupils with other special educational needs and difficulties, their parent-reported anxiety symptoms eased over time while remaining high throughout for autistic pupils. There were no differences in mental health and wellbeing between caregivers of autistic pupils and those with other special educational needs and difficulties. Here, we used parents’ written descriptions of their own and their child’s mental health during the first 6 months of COVID-19 to explore these linked findings in greater depth. We identified strong evidence of worry and distress for all, but most prominently autistic children and young people. Our finding that worry and distress declined over time for pupils with other special educational needs and difficulties, but not for autistic pupils, was supported and we observed a few differences between caregivers. We also found evidence of wellbeing throughout the sample, and examples of some (mainly autistic) pupils benefitting from a reduction in demands (e.g. going to school). This has implications for our understanding of the school experience for autistic pupils. Findings suggest that the mental health of autistic children and young people may have been disproportionately affected during the first 6 months of COVID-19 and that careful consideration of optimal support, from both health and education perspectives, is vital.

We know that autistic children and young people are at substantially increased risk of experiencing mental ill health, including depression (Hudson et al., 2019) and anxiety (van Steensel et al., 2011). We also know that autism is characterised by social and communication challenges and by rigid and repetitive behaviour, interests and activities (American Psychiatric Association, 2013). This profile suggests that the COVID-19 pandemic may have affected the mental health of autistic young people differently to how it has affected neurotypical young people and those with other special educational needs and disabilities (SENDs). Understanding the impact of COVID-19 on autistic young people is, therefore, essential to the design of optimal health and education support as we move forward.

On 23 March 2020, the United Kingdom went into lockdown for the first time in the pandemic, with schools closed to all pupils other than the children of key-workers and those who were vulnerable. Although some young people with autism and other SENDs had the option of attending school as vulnerable pupils, many parents chose to keep them at home. Schools reopened to some year groups in early June and to all pupils in September 2020. This period of disruption may have been particularly challenging for autistic young people who often rely heavily on carefully established routines. Indeed, studies based in Ireland (O’Sullivan et al., 2021) and Portugal (Amorim et al., 2020) have found that disruption to routine during COVID-19 was a key driver of increased anxiety for autistic young people. We also know that parents/carers of autistic young people are likely to have poorer mental health than parents of neurotypical children (Hoffman et al., 2009) or those with other SENDs (Pisula, 2007). The additional caring and home-schooling responsibilities that came with lockdown, alongside COVID-related anxieties, have been found to be particularly challenging for parents of young people with SENDs including autism (Lee et al., 2021). There is some cross-sectional evidence of significant mental health concerns among young people with autism and other SENDs, and in some cases their parents/carers, in the United Kingdom during the first lockdown (Asbury et al., 2021; Banerjee et al., 2021; Nonweiler et al., 2020). There is also evidence that social isolation was a particular trigger for poor mental health and wellbeing among autistic individuals and their parents (Pellicano et al., 2021). However, it is important to note that none of these studies were longitudinal and so they were unable to explore change and stability as the pandemic progressed.

Autistic children and young people have been found to experience anxiety in both similar and different ways to those without autism, and for that anxiety to be triggered and moderated by different factors (Halim et al., 2018; Wood & Gadow, 2010). For example, in a study of autistic children and young people aged 7–17 years, Kerns et al. (2014) found that atypical anxiety symptoms (i.e. symptoms that are inconsistent with the *Diagnostic and Statistical Manual of Mental Disorders* (5th ed.; DSM-V anxiety criteria)) were triggered by both traditional (i.e. DSM-V consistent) anxiety and autism symptoms. These downstream effects manifested in autism-specific anxiety symptoms including unusual, specific phobias, social fearfulness, anxiety around routines and compulsive, ritualistic behaviour. This may have been particularly relevant during COVID-19, at a time when individuals in many households were likely to experience unusual stressors, changes to routine and a lack of certainty. Low tolerance for uncertainty in the autistic community (South & Rodgers, 2017) may have been particularly important in the context of a global pandemic characterised by lockdowns and a reduction in autonomy for all. It is important to note that autistic people have also been found to experience other domains of mental ill health differently to non-autistic people, including depression (e.g. Bitsika & Sharpley, 2015) and eating disorders (e.g. Huke et al., 2013). This means it is important to compare the mental health experiences of autistic individuals with those of non-autistic individuals during COVID-19.

This study had both quantitative and qualitative research strands, and the quantitative findings are presented in Part 1 of this pair of articles (Toseeb & Asbury, 2022). In brief, the quantitative data showed that autistic young people showed higher levels of parent-reported anxiety than those with other SENDs. Furthermore, anxiety decreased between March and October 2020 for young people with other SENDs but not for those with autism. Within the autistic group, it was noted that parent-reported anxiety was higher for older children and girls than it was for younger children and boys. Those with autism also showed higher levels of depression than those other SENDs, but this pattern remained stable over time, with no decrease for either group. Once again, older children and girls showed higher levels of depression, as did pupils in mainstream rather than special education. Parents showed very similar levels of psychological distress and wellbeing whether their child had autism or another SEND and this pattern remained stable over time. However, a significant main effect of household income was noted in both groups, with those in lower-income households reporting higher levels of psychological distress and lower wellbeing.

We used qualitative data to: (1) provide a rich description of how parents described the impact of COVID-19 on their own and their children’s mental health and (2) investigate our quantitative findings in greater depth. We asked the following:

1. How did parents of autistic children and young people describe the effects of the COVID-19 pandemic on their own mental health and that of their children from the beginning of the first COVID-19 lockdown until after school return, 6 months later?
2. To what extent did responses from parents of autistic children and young people differ from those of children and young people with other SENDs?
3. To what extent can parents’ qualitative responses explain the trends observed from quantitative data analysis in Part 1 of this study (Toseeb & Asbury, 2022)?

A qualitative approach is particularly well suited to addressing these questions because, by giving participants the opportunity to comment freely on their experiences we gain access to their personal explanations for why they felt the way they did, and why they believe their children felt and behaved the way that they did in this situation. We also enrich our understanding of what living through a pandemic with a child with autism or other SENDs was like – for both child and parent (from the parent’s point of view) – in terms of tangible and fine grained experiences, something that broad-brush quantitative measures cannot provide. This enhanced understanding may form a useful evidence base for developing hypotheses about the mechanisms that can explain associations between this experience and mental wellbeing in autistic children and young people and their parents, and point towards optimal support mechanisms.

## Method

### Ethics

The study was approved by the Education Ethics Committee at the University of York, UK (reference 20/05). Parents/carers of autistic young people and those with other SENDs provided informed consent electronically.

### Participant recruitment and study design

Parents/carers of 517 autistic young people (75%) and other SENDs completed online questionnaires between 22 March 2020 and 10 October 2020. Of these, 478 provided qualitative data for the analysis reported here (282 at Time 1, 211 at Time 2, 104 at Time 3 and 183 at Time 4). Data were also gathered from parents of 95 non-verbal and minimally verbal children and young people, but these participants were excluded from the current analysis and their data will be analysed and presented separately. This decision was taken because the current analysis is closely tied to the quantitative analysis presented in Toseeb and Asbury (2022) and quantitative mental health data were not gathered from parents/carers of minimally verbal or non-verbal children and young people because the measures used were not appropriate for them. Sample demographics, divided by timepoint, are presented in Part 1 (Toseeb & Asbury, 2022). Young people with a broad range of SENDs were included and full details are provided in Toseeb and Asbury (2022).

During 2020, data were collected at four timepoints: Time 1 (T1: 23 March to 22 April), Time 2 (T2: 23 April to 22 May), Time 3 (T3: 23 May to 22 June) and Time 4 (T4: 29 September to 10 October). Parents took part at one or more timepoints. Those who took part at a previous timepoint were invited to take part in all future timepoints. New participants were recruited in each wave of data collection to maintain sample size. This was particularly important for the quantitative analyses presented in (Toseeb & Asbury, 2022). Sample attrition was as follows: T2 – 56% (173 parents took part at T1 but not T2), T3 – 72% (288 parents took part at T1 or T2 but not T3) and T4 – 63% (326 parents took part at T1, T2 or T3 but not T4). There were 260 (50%) participants who only took part at one timepoint, 158 (31%) who took part at two timepoints, 53 (10%) who took part at three timepoints and 46 (9%) who took part at all four timepoints.

### Measures

Parents/carers were asked a single open-ended item about their own and their child’s mental health at all four timepoints. The wording of the item at T1 was ‘Please describe in your own words how the Coronavirus outbreak is affecting your mental health and your child’s mental health’. The wording at T2–4 was amended slightly to elicit responses related to the preceding month ‘Please describe in your own words how the Coronavirus outbreak has affected your mental health and your child’s mental health in the last month’. Parents/carers were provided with a free text box to input their responses. No word limit was imposed and the average word count for responses was 65 at T1, 72 at T2, 57 at T3 and 61 at T4.

### Analysis and coding

There are ‘no cast iron rules or procedures’ (Neale, 2019, p. 108) for longitudinal qualitative analysis (Saldaña, 2003). We used a repeated cross-sectional design because we have data from different samples of the population of interest at each timepoint, albeit with many individuals represented at more than one timepoint. We conducted content analyses of the qualitative data gathered at each of our four timepoints and summarised them in brief pen portraits (Bengtsson, 2016) in order to gain a bird’s eye view of what changed over time. These individual content analyses highlighted the most prevalent or new issues described by participants at each timepoint in relation to their mental health and that of the autistic young people and those with other SENDs in their care. Our longitudinal analysis followed on from this and focused on how participants’ self-reported mental health changed between T1 and T4 (Grossoehme & Lipstein, 2016). It was conducted in line with Saldaña (2003)’s 16 questions for longitudinal qualitative research. These questions are clustered in three groups: framing questions, descriptive questions and analytic/interpretive questions. The five framing questions are designed to anchor the data in the context of the time in which it was gathered and include ‘What is different from one pond or pool of data through the next?’ and ‘When do changes occur through time?’ These are followed by seven descriptive questions which build on the dynamic time frame established by the framing questions and inform the interpretive questions which follow. They are ‘What?’ questions, such as: ‘What increases or emerges through time?’ and ‘What remains constant or consistent through time?’ Finally, four analytic and interpretive questions are asked of the data, including ‘Which changes interrelate through time?’ and ‘What is the through-line of the study?’

At T1, data were coded inductively by a single coder and, after discussion between the authors, agreed codes were documented in a codebook. All coding was manifest rather than latent as we were interested in representing participants’ actual responses rather than exploring underlying ideas or assumptions. Beyond T1, both deductive and inductive approaches were used; the codes applied to the initial data set were used (deductive) and additional inductive codes were added as needed. When new codes were identified, previous timepoints were re-visited. For instance, if a new code was applied at T3, the authors went back through the (already coded) T1 and T2 data to check for meaning units where it may be appropriate to apply the new code. Code definitions were also re-reviewed iteratively, on the basis of the new data, to ensure shared understanding at all times. In this way, it was ensured that the coding for all four timepoints was comparable. Although T1 data had been previously coded by a team of researchers (Asbury et al., 2021), it was re-coded for this study because of differences in the sample (i.e. additional participants and the exclusion of non-verbal and minimally verbal children and young people from the current study), and in light of revisiting the data on the basis of codes added at later timepoints. In reporting participants’ words, some minor typos or grammatical errors have been corrected for clarity, where doing so does not impact the meaning of the quotation. These codes were then clustered into categories at each timepoint, as described above.

For the current analysis, we only included codes that were applied at least 15 times at one or more timepoints and which were subsequently clustered in one of the three primary categories identified in the data (see section ‘Results and discussion’). In this way, 87 codes in the codebook were reduced to a more manageable 20. Data from every fifth participant in each data set (20% of each sample) at each timepoint was blind-coded by a second rater and Fleiss’ kappa was calculated to assess the inter-rater reliability of the coding.

It can be seen in Table 1 that coding reliability ranged from moderately reliable (*kappa* = 0.60–0.79) through to almost perfectly reliable (*kappa* = 0.90–1.00).

**Table 1.** Inter-rater reliability at all four timepoints, organised by category.

Code	Time 1	Time 2	Time 3	Time 4
Worry				
Anxiety (P)	0.79	0.88	0.77	0.87
Anxiety (C)	0.74	0.79	0.86	0.78
Fear (P)	0.85	0.79	1	1
Fear (C)	0.91	0.84	0.1	0.79
Concern for child’s future (P)	0.81	0.64	0.64	1
Sleep (C)	0.66	0.79	1	1
Won’t go outside (C)	1	1	1	0.79
Psychological Distress				
Low mood (P)	0.90	1	1	0.72
Low mood (C)	1	0.84	1	0.65
Deteriorating mental health (P)	0.65	1	1	1
Deteriorating mental health (C)	0.92	1	0.77	1
Challenging behaviour (C)	0.74	0.79	1	0.91
Negative behaviour change (C)	0.91	0.76	0.77	1
Wellbeing				
No/low impact (P)	1	1	0.83	1
No/low impact (C)	0.74	0.79	1	0.79
Positive emotions (P)	1	0.88	0.64	0.79
Positive emotions (C)	0.84	0.63	0.64	1
Social media helps (C)	1	1	1	1
Positive behaviour change (C)	0.91	0.76	0.77	1

P: parent; C: child.

### Community involvement

There was no community involvement in this study from the autistic community. A member of the research team, who was centrally involved in the design of the study and study questions, is a parent of a child with autism and one other SEND.

## Results and discussion

**Figure 1.**
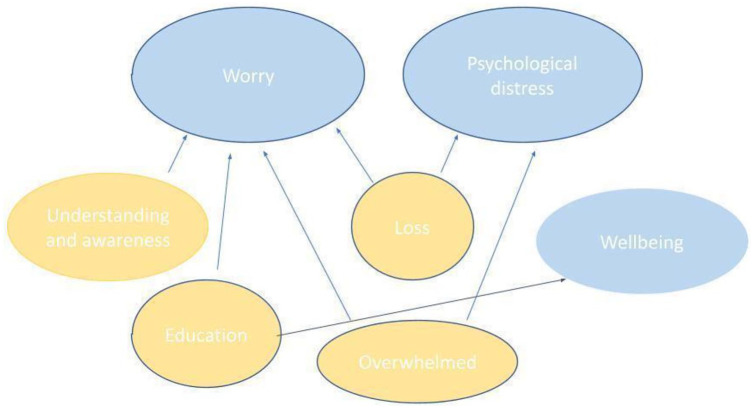
Primary and contributing categories.

In our cross-sectional analyses, we developed seven categories that were present, to a greater or lesser degree, at all four timepoints. We designated three of these as *primary categories* which directly address this study’s research questions about mental health: (1) worry, (2) psychological distress and (3) wellbeing. The remaining four were *contributing categories*: (4) loss, (5) understanding and awareness, (6) overwhelmed and (7) education, because we interpreted them as contributing to the worry, psychological distress or wellbeing that participants described (Figure 1).

In order to be able to explore the data in sufficient depth, we present only the three *primary categories* here, drawing upon the contributing categories to add insight where appropriate.

Table 2 shows how many times each primary category code was applied at each timepoint, and what proportion of each sample (autistic compared to other SENDs) described a situation that was relevant to the code.

**Table 2.** Code frequencies at all four timepoints, organised by category.

Category (code)	Time 1 (*N* = 282)			Time 2 (*N* = 211)			Time 3 (*N* = 104)			Time 4 (*N* = 183)		
	All	ASD	SEND	All	ASD	SEND	All	ASD	SEND	All	ASD	SEND
Worry												
Anxiety (P)	107 (38%)	77 (34%)	30 (52%)	60 (28%)	49 (29%)	11 (28%)	21 (20%)	12 (16%)	9 (29%)	47 (26%)	39 (28%)	8 (18%)
Anxiety (C)	86 (30%)	75 (33%)	11 (19%)	65 (31%)	50 (29%)	15 (38%)	21 (20%)	18 (25%)	3 (10%)	56 (31%)	41 (30%)	15 (33%)
Fear (P)	25 (9%)	21 (9%)	4 (7%)	6 (3%)	5 (3%)	1 (3%)	2 (2%)	2 (3%)	–	9 (5%)	5 (4%)	4 (9%)
Fear (C)	33 (12%)	28 (13%)	5 (9%)	26 (12%)	24 (14%)	2 (5%)	2 (2%)	1 (1%)	1 (3%)	5 (3%)	5 (4%)	–
Concern for child’s future (P)	31 (11%)	27 (12%)	4 (7%)	10 (5%)	9 (5%)	1 (3%)	5 (5%)	3 (4%)	2 (6%)	3 (2%)	3 (2%)	–
Sleep (C)	11 (4%)	11 (5%)	–	15 (7%)	15 (9%)	–	4 (4%)	4 (5%)	–	3 (2%)	2 (1%)	1 (2%)
Won’t go outside (C)	7 (2%)	7 (3%)	–	30 (14%)	28 (16%)	2 (5%)	6 (6%)	6 (8%)	–	6 (3%)	6 (4%)	–
Psychological distress												
Low mood (P)	21 (7%)	16 (7%)	5 (9%)	18 (9%)	13 (8%)	5 (13%)	8 (8%)	6 (8%)	2 (6%)	18 (10%)	16 (12%)	2 (4%)
Low mood (C)	22 (8%)	17 (8%)	5 (9%)	18 (9%)	15 (9%)	3 (8%)	6 (6%)	3 (10%)	3 (10%)	10 (5%)	8 (6%)	2 (4%)
Distress (C)	21 (7%)	18 (8%)	3 (5%)	16 (8%)	13 (8%)	3 (8%)	3 (3%)	2 (3%)	1 (3%)	13 (7%)	11 (8%)	2 (4%)
Deteriorating mental health (P)	16 (6%)	15 (7%)	1 (2%)	14 (8%)	14 (10%)	3 (8%)	8 (8%)	5 (7%)	3 (10%)	14 (8%)	12 (9%)	2 (4%)
Deteriorating mental health (C)	13 (5%)	12 (5%)	1 (2%)	20 (9%)	18 (11%)	2 (5%)	8 (8%)	6 (8%)	2 (6%)	17 (9%)	14 (10%)	3 (7%)
Challenging behaviour (C)	40 (14%)	36 (16%)	4 (7%)	26 (12%)	23 (13%)	3 (8%)	6 (6%)	3 (4%)	3 (10%)	4 (2%)	2 (1%)	2 (4%)
Negative behaviour change (C)	40 (14%)	36 (16%)	4 (7%)	30 (14%)	26 (15%)	4 (10%)	16 (15%)	13 (18%)	3 (10%)	8 (4%)	7 (5%)	1 (2%)
Wellbeing												
No/low impact (P)	19 (7%)	13 (6%)	6 (10%)	11 (5%)	9 (5%)	2 (5%)	8 (8%)	5 (7%)	3 (10%)	16 (9%)	12 (9%)	4 (9%)
No/low impact (C)	24 (9%)	12 (5%)	12 (21%)	9 (4%)	7 (4%)	2 (5%)	4 (4%)	4 (5%)	–	22 (12%)	15 (11%)	7 (16%)
Positive emotions (P)	24 (9%)	19 (8%)	5 (9%)	21 (10%)	11 (6%)	10 (25%)	12 (12%)	8 (11%)	4 (13%)	15 (8%)	10 (7%)	5 (11%)
Positive emotions (C)	39 (14%)	29 (13%)	10 (17%)	49 (23%)	40 (23%)	9 (23%)	22 (21%)	12 (16%)	10 (32%)	23 (13%)	17 (12%)	6 (13%)
Social media helps (C)	1 (0%)	1 (0%)	–	17 (8%)	13 (8%)	4 (10%)	1 (1%)	–	1 (3%)	–	–	–
Positive behaviour change (C)	20 (7%)	17 (8%)	3 (5%)	15 (7%)	13 (8%)	2 (5%)	5 (5%)	3 (4%)	2 (6%)	–	–	–

SEND: special educational need and disability; P: parent; C: child.

Values are the number of responses for which the code was used. The % refers to the percentage of times the code was used as a function of how many participants provided a response at the corresponding timepoint.

This gives us a broad-brush overview of the issues that dominated responses at each timepoint, and how their prevalence differed between families with an autistic young person compared to families with a young person with SENDs other than autism.

### Worry

Our ‘worry’ category represents data that describes and reflects on participants’ feelings or observations of anxiety or worry. Worry was the dominant theme in the data at almost all timepoints, with anxiety being coded in 20%–38% of responses, as shown in Table 2. As one parent put it: ‘I cannot see the end of the tunnel and it is hard to support my children’s fears when it is already difficult to manage my own anxiety’ (P161/T2/Autism).

Participants described symptoms of anxiety in their children that ranged from obsessive handwashing through to epileptic seizures, and included agoraphobia, violence and aggression, self-harm, tics, stimming, sensory issues, breath holding, hand or nail biting, refusing to open windows or leave the house, not sleeping, not eating and nightmares. At T4, one parent said: ‘he believes he will die . . . his brain is telling him to drink the cleaning products and then he will die’ (P485/T4/Autism). Similar examples of severe levels of worry among young people were evident at all four timepoints. It is also important to note, however, that some parents reported a reduction in worry for their children. In general though, parents reported high levels of anxiety in their children that they saw as being directly caused or exacerbated by COVID-19 and lockdown.

As well as describing their children’s worries, parents expressed a great deal of worry themselves. One of their major sources of worry regarded their children falling behind in education. ‘I worry that I’m not doing home ed correctly and that my son will fall even further behind at school due to my failings’ (P237/T1/Autism). Parents and carers also expressed concern about how difficult it would be to get young people back to school after lockdown, with some reporting that they could only manage their child’s worry by not asking them to engage with schoolwork. Some worried about bringing the virus home from work, as is likely to have been the case for many parents working outside the home. However, these concerns were often specific to being the parent of a child with additional needs: ‘I was fine until the government implemented the who lives who dies policy’ (P241/T1/Autism). In other cases, concerns related to who would look after their children if they were to get COVID and die. There was evidence of this level of worry for a meaningful proportion of parents/carers at all four timepoints.

Using Saldaña’s (2003) questions as our framework for longitudinal inquiry, we asked how participants’ worries changed between March/April and October 2020. We noted that T1 – just after the United Kingdom went into its first lockdown and the reality of a global pandemic hit – was the peak for worry-related codes in both parent and child data. We noted a slight decline in the prevalence of these codes by T2, a more marked dip by T3 and then a spike at T4 when young people had mostly returned to school. At T1, parents reported that their children’s anxiety was triggered by the abrupt change of routine, fear of themselves or a loved one becoming ill and overly literal interpretations of social distancing rules. For example, one parent said her son was: ‘scared to venture out because he thinks he will get in trouble from the police, even if he goes in the garden’ (P77/T1/Autism). In some cases, this was exacerbated by young people being unable to understand why these sudden changes were occurring. ‘I think she was confused and a bit scared although she couldn’t explain it herself’ (P43/T1/SEND). Parents’ own worries at T1 most commonly related to their children’s education and to issues such as news about Do Not Resuscitate orders for those with disabilities, and availability of food for young people with a limited diet. There was a sense of shock in the T1 responses that had settled somewhat by T2 when, although many causes for concern were the same, some people had begun to adjust. However, by T2, a growing number of parents expressed concern that they were not doing a good enough job of home education and that learning at home was causing high levels of anxiety for their children. At T3, there was evidence of parent and child worry, driven by similar factors as Times 1 and 2, but the prevalence was noticeably reduced, and most examples were less extreme. There were signs that many participants had accepted or settled into the situation and were coping as well as they could: ‘my daughter was initially very nervous to even leave the house but is starting to overcome this now’ (P45/T3/SEND). It is important to note that this was not the case for everybody though: ‘His anxiety has been very high and he’s had many violent meltdowns’ (P224/T3/Autism).

We observed a spike in worry at T4 when school re-openings triggered high levels of anxiety, mainly for young people but also for some parents. In some cases, participants said young people were worried because they did not want to go to school or found the return difficult. Others, by contrast, wanted to be there but had fears around the safety of the environment and the likelihood of things changing again. In short, we saw high initial levels of parent and child anxiety which by T2 seemed to have become embedded, and slightly reduced in some families. Both the prevalence and severity of anxiety decreased at T3 but by T4, when schools had reopened levels of worry had increased substantially, although not back to T1 levels. This may have been because the re-opening of schools was less worry-inducing for parents, overall, than their closing had been and because reactions among young people were mixed. Overall, the qualitative data mirrored the quantitative findings reasonably accurately, although we should note that we do not have anxiety-specific quantitative data for parents (anxiety symptoms are included in the measure used for psychological distress).

We also asked whether patterns of worry differed for young people with a diagnosis of autism and those with other diagnoses, as indicated in the quantitative data, and whether there was any difference between the parents/carers of these two groups. Findings generally aligned well with the quantitative data. The qualitative data showed a few differences between the two groups of parents, as was also the case in the quantitative data. The quantitative data also showed that autistic young people showed higher levels of anxiety at all timepoints and that, unlike those with other SENDs, their anxiety did not decrease over time. This was broadly supported by the qualitative data. We noted a striking decrease in worry for young people with SENDs by T2, but not for autistic young people, perhaps reflecting relative success in adjusting to the new circumstances. This also aligns well with the literature on the role of intolerance of uncertainty in autistic anxiety (Halim et al., 2018; South & Rodgers, 2017). There were signs of a decrease in worry-related codes for all at T3 (not seen in the quantitative data), and an increase for all at T4 that was primarily driven by autistic young people. Interestingly, Table 2 shows that proportionately more parent anxiety codes were applied to data from parents of children with SENDs other than autism at T1. While the reason for this is uncertain, one possible explanation is that more parents of autistic children benefitted from an initial reduction in their child’s anxiety, possibly as a result of reduced social and educational demands, and therefore felt a little less anxious themselves. That said, autistic young people were still clearly the more anxious group.

### Psychological distress

The psychological distress category represents data that describes and reflects on feelings such as sadness, low mood or despair and behaviour such as crying or acting out in ways that could be interpreted as representing distress. Codes related to psychological distress were less prevalent in the data than codes related to worry. However, it is important to note that these two categories, while distinct, cannot be viewed as entirely unrelated. There were signs in the data that distress and its various manifestations were often triggered by anxiety, even if that was not explicitly stated by participants. It was notable that low mood codes were applied in 7%–10% of cases for parents and 5%–10% of cases for young people, with distress and deteriorating mental health showing a similar prevalence. Interestingly, the prevalence of challenging behaviour and negative behaviour change was higher – particularly at T1 and T2 where it was around 14% – suggesting that this, rather than depressive symptoms, was the primary way in which young people showed their psychological distress.

For parents/carers, distress was triggered by internal factors including anxiety, feeling overwhelmed and exhausted, challenging behaviour from their child, guilt about how well they were supporting their child, loneliness and, for some, a loss of purpose. For example, ‘I’m feeling very low and hopeless and don’t know what’s going to happen to her in the future because she’s regressing so badly’ (P307/T1/Autism). External triggers for psychological distress included the sudden change of routine, a lack of respite, experiences of violence and aggression and the demands of home education. ‘Our entire support network has been taken away from us, including family. I am a single parent of twins, both of who have complex learning difficulties, and the thought of having to manage alone fills me with dread’ (P194/T1/Autism). This reflects Pellicano et al.’s (2021) findings regarding the detrimental impact of social isolation on mental health and wellbeing among autistic people and their families. One participant pointed out the overwhelming nature of lockdown with a child with additional needs, noting a role as a carer that is not required of other parents: ‘It is exhausting trying to be parent, carer and teacher’ (P283/T2/Autism). There were no notable differences between the parents of those with autism and those with other SENDs in terms of the regularity with which they talked about psychological distress, or its severity.

The psychological distress reported for young people was primarily displayed through challenging behaviour or negative behaviour change and was exacerbated by a lack of support. For example, ‘my child is struggling and this can lead to violent threats and abuse that we cannot get away from’ (P389/T1/Autism). There were some signs of low mood in the data ranging from mild depressive symptoms, ‘Lacking motivation and enjoyment for things’ (P369/T3/SEND) through to suicidal ideation and suicide attempts:

The lockdown had a devastating impact on our son to the extent that he became very close to being sectioned. The rug was pulled completely from under his feet and he just could not cope, became extremely violent, tried to take his life, and became quite manic. He is slowly recovering but is hardly attending school and when he does he does not cope. (P179/T4/Autism)

It was notable that the severity of distress, including low mood, described by participants was generally higher for autistic young people than it was for those with other SENDs. Some of this aligns well with Halim et al.’s (2018) finding that one way of coping with the experience of shutting or melting down is to engage in self-injurious behaviour as a way of re-setting. The distress of most young people with SENDs was triggered by common factors including missing friends and routine. By contrast, the self-harm, suicidal ideation, food refusal and meltdowns described for some autistic young people represented a generally more severe level of distress: ‘My son is crying all the time and said that he cannot handle all this, he has started talking to people who are not there’ (P165/T1/Autism). It is important to note that while such extreme reactions were identified in a minority of the sample, there was a broad spectrum of responses, as would be expected and as illustrated by our Category 3 (wellbeing) analysis below. Across both groups, there were signs that low levels of understanding and awareness affected anxiety and distress, but not always in the same direction. While a lack of understanding triggered high levels of distress in some young people, it served as a protective factor for others. For example, at T1, one parent said: ‘She doesn’t understand why things have changed so instead she’s become aggressive. She’s broken a window and an oven’ (P120/T1/Autism); while another, by contrast, said: ‘I don’t think my child has any idea anything bad is happening’ (P139/T1/Autism).

Our longitudinal analysis identified elements of both stability and change over time. At T1, we noted high levels of distress among both parents and young people that were more prevalent and severe for autistic young people than those with other SENDs, but which showed no differences for parents/carers. Much of this distress was triggered by an abrupt change of routine and widespread uncertainty. While distress was maintained at a similar level in T2, there was evidence of two specific drivers for much of it. The first, and most widespread, related to home education which was completely unacceptable to some of these young people, leading to high levels of distress and challenging behaviour, and to guilt and feelings of failure for parents: ‘My child began to recover from the trauma of school since schools were closed . . . Since Easter her mental health is deteriorating to the point of feeling suicidal due to the schoolwork making her overwhelmed’ (P565/T2/Autism). The second driver related specifically to those young people who had needed mental health support prior to lockdown, and the sudden withdrawal of that support:

A couple of months prior to going into lockdown [child] was expressing suicidal thoughts. School had arranged some counselling for him but this hasn’t taken place and I feel we have been abandoned. He is still feeling very low and still expressing suicidal thoughts. It feels like everything is focused on Covid-19 and there is no support for anything else. (P39/T2/Autism)

These young people and their families were made particularly vulnerable during COVID-19 lockdowns and, in almost all cases, had an autism diagnosis.

There was a change of tone in participants’ responses at T3 that was perhaps indicative of fatigue and resignation setting in, and this was the time at which differences between the two groups were the least pronounced. Although we did not see change in the prevalence of low mood codes, there was a reduction in challenging behaviour codes, and in the type of overt distress described above. Home education was still triggering outbursts in some young people, but this was less prevalent than at T2, perhaps because more had returned to school or because some families had given up trying in order to reduce demands on their children and make their home life more manageable. Overall, responses at T3 were characterised by lethargy, ‘My mental health has deteriorated but am now medicated’ (P374/T3/SEND). Perhaps surprisingly, the return to school by T4 did not increase or decrease distress for either group very much, or for parents – which was not the case for worry where we observed a clear spike when young people returned to school. Many participants still sounded rather ground down, partly because even those who were pleased about school re-openings were struggling with uncertainty and the likelihood of further hits to routine.

While going back to school was a positive experience for many young people, more of those with autism than other SENDs struggled with it and in some cases returned to an environment in which they were bullied or felt they did not fit in:

My son has struggled with returning to school and doing homework. His antidepressant medication dose has been increased and this seems to have taken the edge off a bit since then but he feels that the only way to help him feel better is to stop making him go to school. And of course now he has had experience of life without school being better. (P312/T4/Autism)

Also, while many parents were relieved that their children could return to school some struggled with anxiety, fear, uncertainty and, for a minority, loneliness and a loss of self-worth after several months in which they had felt busier and more needed than before. In short, the picture was complex and low-level distress was evident in participants’ responses.

Overall, these findings support and shed light on findings from our quantitative data. Specifically, we observed that the prevalence and severity of parents’ psychological distress did not seem to differ by group or, other than a dip in energy at T3, to change over time. While there were cases in which it was clear that household income was a stressor, not enough participants talked about this for us to be able to develop a clear view on whether those from lower-income households experienced higher distress. The qualitative data supported the finding that autistic young people experienced higher levels of psychological distress than young people with other SENDs at all timepoints, although there was some suggestion that the difference was less pronounced at T3. It was also evident that older children and girls were more severely affected than younger children and boys. It was less clear whether those who attended mainstream schools were more severely affected than those in special schools and, if so, why that was the case.

### Wellbeing

This ‘wellbeing’ category draws together evidence of increased wellbeing, including positive emotions and a calmer home environment, as a result of COVID-19-related changes. A substantial minority of participants reported mental health and wellbeing benefits for their children, and to a lesser extent themselves, as a direct result of not having to go to school during COVID-19 lockdowns. As one parent put it: ‘he no longer has the daily torture of going to school’ (P71/T1/Autism). Codes associated with wellbeing were more prevalent in our data than those associated with psychological distress, although less prevalent than those associated with worry.

Participants reported good levels of positive emotions for young people at all timepoints, but particularly at Times 2 and 3 when almost one-quarter of the codes applied to the data reflected positive child emotions:

My son is loving being at home. He hasn’t got dressed since his last day at school, his appetite has improved and he’s doing some of his work with support because now I am his 1:1. I’m available for every piece of work to prompt him when he forgets what he’s doing or his attention wanders. He’s loving not going out (P460/T2/Autism).

It is clear that the removal of school from some young people’s daily lives made a huge and positive difference to them, largely by reducing demands. It was common for home to be referred to as a safe and happy place. It was notable that improvements in physical health, as well as wellbeing, were described. ‘My daughter had very bad headaches when at school and we had been referred to hospital. These have completely ceased since lockdown’ (P210/T2/Autism). In summary, the removal of the requirement to attend school appears to have been a substantial driver of wellbeing for some young people. For some, however, this was contingent on not being pressured to do any schoolwork at all at home, with clear implications for post-lockdown educational recovery.

Wellbeing codes were less prevalent in the parent data than the child data but, nonetheless, approximately 10% of codes related to parents’ positive emotions, and this was stable across the four timepoints. For example, ‘I have never been calmer or more content’ (P293/T1/Autism). There were no major wellbeing differences observed between timepoints, or between the parents of autistic young people compared to those with other SENDs, supporting our quantitative findings. Furthermore, some participants acknowledged the importance of material comfort and security in being able to cope well with lockdown. For example, ‘We are very fortunate. We have isolated hard but had the means to do so in comfort’ (P401/T3/SEND). The primary wellbeing benefit for parents came from having children who were calmer, happier and less stressed than usual, and there were many descriptions of reductions in meltdowns, aggressive behaviour and anxiety. ‘My child hates going to school and would have daily meltdowns on arrival at the school gates. We are not having any of these meltdowns’ (P499/T1/Autism). Parents also benefitted from reduced demands on themselves as well as on their children. For example,

I feel less under pressure to battle with her to go to the supermarket or to cinema or park etc as she always finds these a struggle noisewise and she doesn’t like other people being near her, so being isolated has taken away a lot of stress. (P562/T1/Autism)

In terms of how the pattern of wellbeing codes and comments changed over time, we saw that wellbeing benefits were evident from T1 and increased at T2, remaining stable at T3. The most notable change came at T4, when most young people were back at school, and the prevalence of wellbeing codes dropped back to the T1 level (e.g. positive emotions codes made up 8% of parent data and 13% of child data). At T4, some parents were very positive about the wellbeing benefits of going back to school, for example. Even for parents who were positive about this change, however, their pleasure was often tinged with worry about future closures or about whether their child’s mental health and wellbeing would deteriorate again, perhaps explaining the dip in wellbeing codes at this timepoint. Overall, we noted a drop in wellbeing codes at T4, but no difference between autistic young people and those with other SENDs. Looking across all timepoints, aside from the positive evidence that some young people seem to have thrived during lockdowns, there is the clear, negative, underpinning message that school is really hard for some of these young people.

### Implications and future directions

These findings add to our understanding of the impact of COVID-19 on autistic young people and their parents/carers and we hope they will be useful in the event of future lockdowns as well as in a future in which COVID-19 becomes endemic. In the event of a future lockdown, these findings suggest ways of managing transitions in and out of school more effectively, and of tailoring home education demands so that they are less anxiety-inducing for both pupils and parents. They also strongly suggest that existing support should be kept in place in whatever form is possible. In the event that we have no further lockdowns but are living with the virus, our participants’ views can be used to inform initiatives around reducing uncertainty for autistic individuals and those who care for them, such as providing very clear guidance around what is and is not allowed when restrictions are in place.

Our findings suggest that autistic young people have been disproportionately affected by COVID-19-related disruption and that, therefore, careful consideration of how they can be supported is vital. Those who have not been able to access the mental health support they had been granted prior to the pandemic should be considered as a particular and pressing priority, and the need for professional mental health support is likely to have expanded to a wider group. Participants’ descriptions of school-related anxiety suggest that much remains to be done to make most schools ‘autism-friendly’ and that, given these young people experience higher levels of anxiety and distress than neurotypical young people and those with other SENDs, this should also be considered a priority.

In regard to parents, our findings suggest that any mental health and wellbeing support for parents/carers can potentially be shared across a range of diagnoses and that parents of autistic young people will not necessarily need separate support.

Since October 2020 there has been a further lockdown, and widespread self-isolation and it is important to consider the ongoing implications of this for the mental health and wellbeing of autistic young people and their families. Future research should address the period beyond October 2020 and gather data from autistic young people and those with other SENDs, as well as from their parents/carers. There would also be value in studying teachers’ perspectives of how successfully autistic young people have made the transition back into school. A qualitative approach can be particularly valuable in adding important details to the understanding we gain from quantitative studies of mental health in these young people, and therefore, mixed-methods research is recommended. Future studies should also incorporate community involvement from the autistic community into their design, delivery, analysis and dissemination (Fletcher-Watson et al., 2021). Not doing so in this study represents a clear limitation of the research.

Finally, it is important to note the degree of diversity in participants’ responses, suggesting that a ‘one size fits all’ approach to supporting autistic young people during the global recovery from COVID-19 is likely to be ineffective, and that a highly personalised approach is likely to be necessary. There are lessons to be drawn from tales of high wellbeing as well as those of high anxiety and distress. The current findings offer insights to policymakers, school leaders, teachers and health and social care professionals who are working to support autistic young people and their families through the remainder of this global pandemic and beyond.
